# Predictive nomogram for postoperative atrial fibrillation in locally advanced esophageal squamous carcinoma cell with neoadjuvant treatment

**DOI:** 10.3389/fsurg.2022.1089930

**Published:** 2023-01-04

**Authors:** Meiqin Fang, Mingduan Chen, Xiaoqiang Du, Shuchen Chen

**Affiliations:** ^1^ Fujian Key Laboratory of Vascular Aging, Fujian Medical University, Fuzhou, China; ^2^ Department of Geriatrics, Fujian Medical University Union Hospital, Fuzhou, China; ^3^ Department of Thoracic Surgery, Fujian Medical University Union Hospital, Fuzhou, China; ^4^ Key Laboratory of Cardio-Thoracic Surgery, Fujian Medical University, Fujian Province University, Fuzhou, China; ^5^ Key Laboratory of Ministry of Education for Gastrointestinal Cancer, Fujian Medical University, Fuzhou, China; ^6^ Fujian Key Laboratory of Tumor Microbiology, Fujian Medical University, Fuzhou, China; ^7^ Department of Radiology, Fujian Medical University Union Hospital, Fuzhou, China

**Keywords:** esophageal squamous carcinoma cell, neoadjuvant treatment, postoperative atrial fibrillation, risk factors, nomogram model

## Abstract

**Background:**

Neoadjuvant therapy following minimally invasive esophagectomy is recommended as the standard treatment for locally advanced esophageal squamous carcinoma cells (ESCC). Postoperative atrial fibrillation (POAF) after esophagectomy is common. We aimed to determine the risk factors and construct a nomogram model to predict the incidence of POAF among patients receiving neoadjuvant therapy.

**Methods:**

We retrospectively included patients with ESCC receiving neoadjuvant chemotherapy (nCT), neoadjuvant chemoradiotherapy (nCRT), or neoadjuvant immunochemotherapy (nICT) following minimally invasive esophagectomy (MIE) for analysis. Patients without a history of AF who did not have any AF before surgery and who developed new AF after surgery, were defined as having POAF. We applied a LASSO regression analysis to avoid the collinearity of variables and screen the risk factors. We then applied a multivariate regression analysis to select independent risk factors and constructed a nomogram model to predict POAF. We used the receiver operating characteristic (ROC) curve, calibration curve, and decision curve analysis (DCA) curve to evaluate the nomogram model.

**Results:**

A total of 202 patients were included for analysis, with 35 patients receiving nCRT, 88 patients receiving nCT, and 79 patients receiving nICT. POAF occurred in 34 (16.83%) patients. There was no significant difference in the distribution of neoadjuvant types between the POAF group and the no POAF group. There was a significant increase in postoperative hospital stay (*p* = 0.04), hospital expenses (*p* = 0.01), and comprehensive complication index (*p* < 0.001). The LASSO analysis screened the following as risk factors: blood loss; ejection fraction (EF); forced expiratory volume in 1 s; preoperative albumin (Alb); postoperative hemoglobin (Hb); preoperative Hb; hypertension; time to surgery; age; and left atrial (LA) diameter. Further, preoperative Alb ≤41.2 g/L (*p* < 0.001), preoperative Hb >149 g/L (*p* = 0.01), EF >67.61% (*p* = 0.008), and LA diameter >32.9 mm (*p* = 0.03) were determined as independent risk factors of POAF in the multivariate logistic analysis. The nomogram had an area under the curve (AUC) of 0.77. The Briser score of the calibration curve was 0.12. The DCA confirmed good clinical value.

**Conclusions:**

Preoperative Alb ≤41.2 g/L, LA diameter >32.9 mm, preoperative Hb >149 g/L, and EF >67.61% were determined as the risk factors for POAF among patients with ESCC. A novel and valuable nomogram was constructed and validated to help clinicians evaluate the risk of POAF and take personalized treatment plans.

## Introduction

Esophageal squamous carcinoma cell (ESCC) is the primary subtype of esophageal cancer (EC) in Asia, especially in China (1). A combination of neoadjuvant therapy and surgery is recommended as the standard treatment for locally advanced ESCC. There is still no consensus on neoadjuvant therapy. Compared with surgery alone, both neoadjuvant chemotherapy (nCT) and neoadjuvant chemoradiotherapy (nCRT) have been confirmed to improve overall survival and disease-free survival (2, 3). The nCRT pattern is recommended as the first choice in the national comprehensive cancer network (NCCN) and Chinese society of clinical oncology (CSCO) guidelines. However, due to unpromising long-term survival and the high distant recurrence incidence, the exploration of novel treatment patterns is necessary. Phase II clinical trials showed that neoadjuvant immunochemotherapy (nICT) had a promising pathological response and manageable adverse events (4, 5).

With the development of minimally invasive esophagectomy (MIE), morbidity and mortality have reduced (6); however, complications (especially pneumonia, anastomotic leakage, and atrial fibrillation) after MIE are still high, and management is still challenging. Among patients with solid cancers, patients with EC had the highest risk of atrial fibrillation [adjusted sub-distribution hazard ratio (HR) 2.69; 95% confidence interval (CI) 2.45–2.95] (7). In patients who underwent esophagectomy, postoperative atrial fibrillation (POAF) was highly associated with postoperative infectious complications (8). A recent meta-analysis showed that the incidence of POAF was 16.5%, and patients with POAF had a higher risk of anastomotic leakage, pneumonia, death, and other adverse events (9). In addition, a 21-year follow-up cohort showed that POAF was associated with poorer long-term survival after esophagectomy (HR 2.99, 95% CI=1.37–6.53). Further, POAF increased the risk of stroke, cognitive decline, and depression, reduced the quality of life, and brought a great burden to patients and the medical system (10).

Previous reports indicated that the application of neoadjuvant treatment contributed to the occurrence of POAF (11). Considering the promotion of neoadjuvant treatment plus esophagectomy, it is of clinical importance to understand the risk factors of POAF among patients receiving neoadjuvant therapy (nCT, nCRT, or nICT). The aim of the present study was to determine the risk factors of POAF among patients receiving neoadjuvant therapy and construct a nomogram model to help clinicians evaluate the risk of POAF and take personalized treatment plans. Another concern was whether POAF was associated with the types of neoadjuvant treatment.

## Materials and methods

### Patient selection

This was a retrospective analysis based on a prospectively collected dataset. The inclusion criteria were as follows: (1) diagnosed with ESCC; (2) clinical stage in the range of II–IVA; (3) receiving nCT, nCRT, or nICT; (4) undergoing radical transthoracic MIE (including robotic-assisted and video-assisted); and (5) without AF before operation. The exclusion criteria were as follows: (1) a history of heart failure or preoperative AF; (2) severe liver and kidney dysfunction; (3) unresectable tumors or metastases during exploratory surgery; (4) cervical EC; and (5) laryngopharyngeal carcinoma-esophagectomy. This study was approved by the ethics committee at Fujian Medical University Union Hospital. In addition, this study was conducted in strict accordance with the Declaration of Helsinki (1964).

### Data collection and definition of variables

The patients' demographic characteristics [sex, age, body mass index (BMI], smoking history, drinking history, preoperative complications, American society of anesthesiologists (ASA) status), preoperative examinations [preoperative albumin, preoperative hemoglobin (Hb), ejection fraction (EF), forced expiratory volume in 1 s (FEV1)], neoadjuvant treatment (types, time to surgery), tumor characteristics (tumor location, pathological grade, pathological T stage, pathological N stage, lymph nodes removed number), surgery (types, surgical time, blood loss), and postoperative information [comprehensive complication index (CCI), hospital stay, thoracic tube stay, hospital expenses] were collected for analysis.

POAF was the primary outcome of this study. Patients without a history of AF who did not have any type of AF before surgery and who developed new AF after surgery, were defined as POAF. The tumor location was divided into upper third, middle third, and lower third. The pathological TNM stage used in this study was the 8th AJCC staging system. Neoadjuvant treatment included nCT, nCRT, and nICT. The CCI was developed based on the Clavien–Dindo classification system to measure the severity of postoperative complications. The calculation was conducted at www.assessurgery.com.

### Statistical analysis

First, we divided the patients into a POAF group and no POAF group. We expressed the continuous data as mean ± standard deviation or median (interquartile range) and the categorical data as numbers (percentages). The comparisons of baseline characteristics and postoperative information were compared. The Student's *t*-test or Mann–Whitney *U* test was used for continuous variables, and the chi-square test or Fisher's exact test was used for categorical variables. The continuous variables were converted into categorical variables according to the optimal cutoff value of the receiver operator characteristic (ROC) curve or clinical experience. Second, due to the relatively large number of variables and to avoid the collinearity of variables, we used the LASSO regression model to screen the variables. The principle of LASSO regression screening the variables is to compress the regression coefficients of each variable in the form of a penalty increase (12). Further, we also conducted cross-validation to verify the Lasso regression model. Third, the risk factors screened by the LASSO regression model were included in a multivariate logistic regression model to further determine the independent risk factors. Four, a nomogram model was constructed based on the screened independent risk factors. We evaluated the predictive ability of the nomogram by ROC and area under the curve (AUC) values. We measured the agreement between predicted and actual results by calibration curves. We further evaluated the clinical value of the nomogram model by decision curve analysis (DCA). We conducted the statistical analysis using R software (version 3.6.3) and Python (version 3.7). The two-sided *p* < 0.05 was considered statistically significant in this study.

## Results

### Comparisons of preoperative characteristics between the POAF and no POAF groups

A total of 202 patients were included for analysis. POAF occurred in 34 (16.83%) patients. There were 35 patients receiving nCRT, 88 patients receiving nCT, and 79 patients receiving nICT. There was a significant difference in age, preoperative ALB, preoperative Hb, EF, and FEV1 between the POAF and no POAF groups (*p* < 0.05). There were no statistically significant differences between the POAF and no POAF groups in BMI, smoking history, drinking history, ASA status, blood loss, surgical time, MIE type, lymph nodes moved number, tumor location, pathological grade, pathological T stage, pathological N stage, neoadjuvant type, left atrial (LA) diameter, and time to surgery (*p* > 0.05). The details of comparisons of baseline characteristics between the POAF and no POAF groups are presented in Table 1.

**Table 1:** Comparisons of demographic and clinicopathological characteristics between the POAF group and no POAF group.

Contents	Variables	Total (*n* = 202)	No POAF (*n* = 168)	POAF (*n* = 34)	*P*
Age (years)	≦62	113 (55.94)	102 (60.71)	11 (32.35)	0.002
	>62	89 (44.06)	66 (39.29)	23 (67.65)	
BMI	≦20.54	57 (28.22)	50 (29.76)	7 (20.59)	0.28
	>20.54	145 (71.78)	118 (70.24)	27 (79.41)	
Smoking history	No	87 (43.07)	71 (42.26)	16 (47.06)	0.61
	Yes	115 (56.93)	97 (57.74)	18 (52.94)	
Drinking history	No	147 (72.77)	122 (72.62)	25 (73.53)	0.91
	Yes	55 (27.28)	46 (27.38)	9 (26.47)	
Hypertension	No	166 (82.18)	144 (85.71)	22 (64.71)	0.004
	Yes	36 (17.82)	24 (14.29)	12 (35.29)	
Diabetes	No	191 (94.55)	160 (95.24)	31 (91.18)	0.34
	Yes	11 (5.45)	8 (4.76)	3 (8.82)	
Coronary heart disease	No	196 (97.03)	164 (97.62)	32 (94.12)	0.27
	Yes	6 (2.97)	4 (2.38)	2 (5.88)	
ASA status	≦2	175 (86.63)	145 (86.31)	30 (88.24)	0.76
	>2	27 (13.37)	23 (13.69)	4 (11.76)	
EF	≦67.6%	104 (51.49)	93 (55.36)	11 (32.35)	0.01
	>67.6%	98 (48.51)	75 (44.64)	23 (67.65)	
FEV1	≦2.38	76 (37.62)	57 (33.93)	19 (55.88)	0.02
	>2.38	126 (62.38)	111 (66.07)	15 (44.12)	
LA (mm)	≦32.9	141 (69.80)	120 (71.43)	21 (61.76)	0.04
	>32.9	61 (30.20)	48 (28.57)	13 (38.24)	
Preoperation alb (g/L)	≦41.2	119 (58.91)	90 (53.57)	29 (85.29)	<0.001
	>41.2	83 (41.09)	78 (46.43)	5 (14.71)	
Post D1 Alb (g/L)	≦31	46 (22.77)	35 (20.83)	11 (32.35)	0.14
	>31	156 (77.23)	133 (79.17)	23 (67.65)	
Preoperation Hb (g/L)	≦149	189 (93.56)	160 (95.24)	29 (85.29)	0.03
	>149	13 (6.44)	8 (4.76)	5 (14.71)	
Post D1 Hb (g/L)	≦138	176 (87.13)	149 (88.69)	27 (79.41)	0.14
	>138	26 (12.87)	19 (11.31)	7 (20.59)	
Time to surgery (day)	≦44	124 (61.39)	99 (58.93)	25 (73.53)	0.11
	>44	78 (38.61)	69 (41.07)	9 (26.47)	
Tumor location	Upper third	22 (10.89)	15 (8.93)	7 (20.59)	0.13
	Middle third	111 (54.95)	95 (56.55)	16 (47.06)	
	Lower third	69 (34.16)	58 (34.52)	11 (32.35)	
Neoadjuvant type	nCRT	35 (17.33)	31 (18.45)	4 (11.77)	0.09
	nCT	88 (43.56)	77 (45.83)	11 (32.35)	
	nICT	79 (39.11)	60 (35.71)	19 (55.88)	
Surgical time (min)	≦300	78 (38.61)	65 (38.69)	13 (38.24)	0.96
	>300	124 (61.39)	103 (61.31)	21 (61.76)	
Blood loss (ml)	≦150	173 (85.64)	147 (87.50)	26 (76.47)	0.09
	>150	29 (14.36)	21 (12.50)	8 (23.53)	
MIE type	Video-assisted	169 (83.66)	143 (85.12)	26 (76.47)	0.21
	Robotic-assisted	33 (16.34)	25 (14.88)	8 (23.53)	
ypG	G0	43 (21.29)	35 (20.83)	8 (23.53)	0.35
	G1	33 (16.34)	31 (18.45)	2 (5.88)	
	G2	47 (23.27)	38 (22.62)	9 (26.47)	
	G3	79 (39.11)	64 (38.10)	15 (44.12)	
ypT	T0	43 (21.29)	35 (20.83)	8 (23.53)	0.89
	T1–2	66 (32.67)	56 (33.33)	10 (29.41)	
	T3–4	93 (46.04)	77 (45.83)	16 (47.06)	
ypN	N0	110 (54.46)	90 (53.57)	20 (58.82)	0.58
	N+	92 (45.54)	78 (46.43)	14 (41.18)	
Lymph nodes moved number	≦38	138 (68.32)	118 (70.24)	20 (58.82)	0.19
	>38	64 (31.68)	50 (29.76)	14 (41.18)	

POAF, postoperative atrial fibrillation; BMI, body mass index; Hb, hemoglobin; Alb, albumin; FEV1, forced expiratory volume in one second; EF, ejection fraction; MIE, minimally invasive esophagectomy; LA, left atrial.

**Figure 1:**
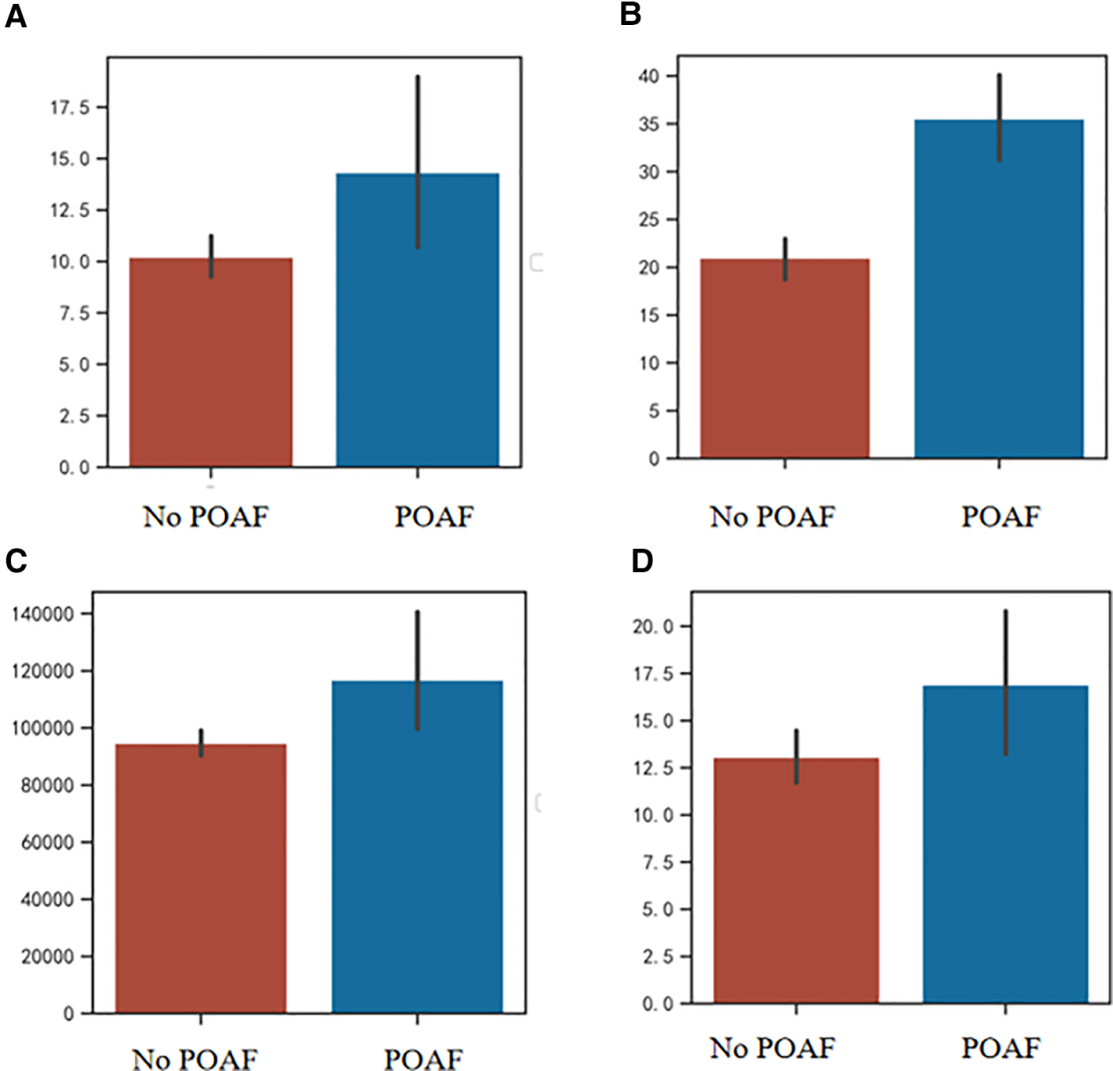
(**A**) Comparison of thoracic tube stay between POAF group and no POAF group; (**B**) Comparison of CCI between POAF group and no POAF group; (**C**) Comparison of hospital expenses between POAF group and no POAF group; (**D**) Comparison of postoperative hospital stay between POAF group and no POAF group. POAF, postoperative atrial fibrillation; CCI, comprehensive complication index.

Compared with the no POAF group, the POAF group had an increase in postoperative hospital stay (median 11 days vs. 10 days), CCI (median 32.00 vs. 22.60), and hospital expenses (median 99707.22 yuan vs. 88916.27 yuan). There was no significant difference in total hospital stay and thoracic drainage tube stays (*p* > 0.05). The details of the comparisons of perioperative outcomes were summarized in Table 2 and presented in Figure 1.

**Table 2:** Comparisons of perioperative outcomes between the POAF group and no POAF group.

Contents	Total (*n* = 202)	No POAF (*n* = 168)	POAF (*n* = 34)	*P*
Thoracic drainage tube stay (days)	10.90 ± 8.02	10.22 ± 6.51	14.33 ± 12.72	0.08
Postoperative hospital stay (days)	10[8,14]	10[8,13]	11[9,27]	0.04
Total hospital stay (days)	16[12,24]	16[12,24]	16[13,35]	0.16
CCI	24.20[8.70,32.00]	22.60[8.70,28.90]	32.00[25.70,44.30]	<0.001
Hospital expenses (RMB)	89,623.27[79,549.69,107,293.68]	88,916.27[79,113.85,103,342.57]	99,707.22[84,003.12,134,154.56]	0.01

POAF, postoperative atrial fibrillation; CCI, comprehensive complication index; RMB, ren min bi.

### Screening predictive factors using LASSO logistic regression analysis

**Figure 2:**
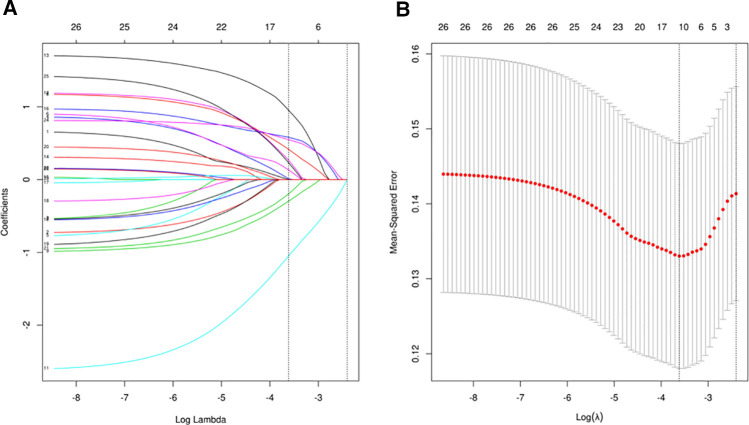
(**A**) The regression analysis of influence factors based on Lasso analysis for variable selection; (**B**) the cross-validation of the regression model.

LASSO regression analysis (Figure 2A) and cross-validation (Figure 2B) were performed for each influencing factor, and the independent variables were further screened. The value with the smallest verification error (*λ* = 0.12) was selected to fit the regression model, and there were 10 variables of the model in total, including blood loss, EF, FEV1, preoperative Alb, postoperative D1 Hb, preoperative Hb, hypertension, time to surgery, age, and LA diameter. Further, multivariate logistic regression, including the above 10 predictive factors, was conducted to determine the independent risk factors. Preoperative Alb ≦41.2 g/L (*p* < 0.001), preoperative Hb >149 g/L (*p* = 0.01), EF >67.61% (*p* = 0.008), and LA diameter >32.9 mm (*p* = 0.03) were determined as the independent risk factors of POAF. The results of multivariate logistic regression are summarized in Table 3.

**Table 3:** Multivariate logistic analysis of postoperative atrial fibrillation.

Predictor	Before selection				After selection			
	*p*	OR	Lower	Upper	*p*	Odds ratio	Lower	Upper
Blood loss ≦ 150 ml	Reference							
Blood loss > 150 ml	0.25	1.90	0.61	5.64				
FEV1 ≦ 2.38 L	Reference							
FEV1 > 2.38 L	0.10	0.45	0.17	1.16				
EF ≦ 67.61%	Reference							
EF > 67.61%	0.03	2.99	1.17	8.31	0.008	3.23	1.40	8.06
Preoperation Alb ≦ 41.2 g/L	Reference							
Preoperation Alb > 41.2 g/L	0.001	0.12	0.03	0.35	<0.001	0.14	0.04	0.37
Postoperation D1 Hb ≦ 138 g/L	Reference							
Postoperation D1 Hb > 138 g/L	0.06	3.55	0.94	13.39				
Preoperation Hb ≦ 149 g/L	Reference							
Preoperation Hb > 149 g/L	0.02	6.46	1.27	33.99	0.01	5.82	1.40	24.23
Hypertension no	Reference							
Hypertension yes	0.10	2.30	0.84	6.25				
Time to surgery ≦ 44 days	Reference							
Time to surgery > 44 days	0.07	0.39	0.13	1.03				
Age ≦ 62 years	Reference							
Age > 62 years	0.08	2.35	0.91	6.39				
LA diameter ≦ 32.9 mm	Reference							
LA diameter > 32.9 mm	0.02	3.33	1.21	9.59	0.03	2.67	1.09	6.64

OR, odds ratio; Hb, hemoglobin; Alb, albumin; FEV1, forced expiratory volume in one second; EF, ejection fraction; LA, left atrial.

### Development and validation of a nomogram model

**Figure 3:**
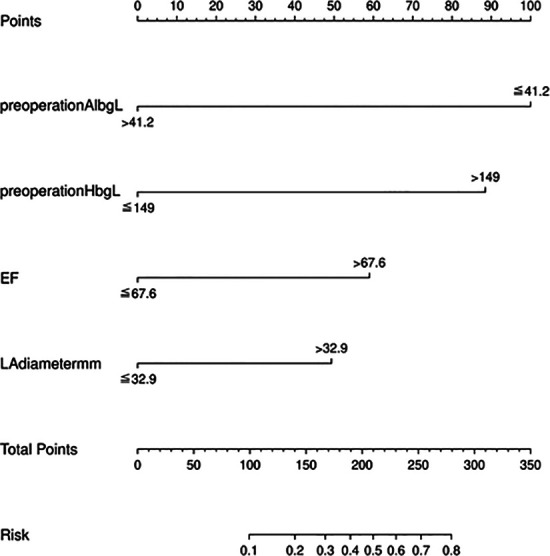
The nomogram model to predict postoperative atrial fibrillation among patients receiving neoadjuvant therapy and minimally invasive esophagectomy.

**Figure 4:**
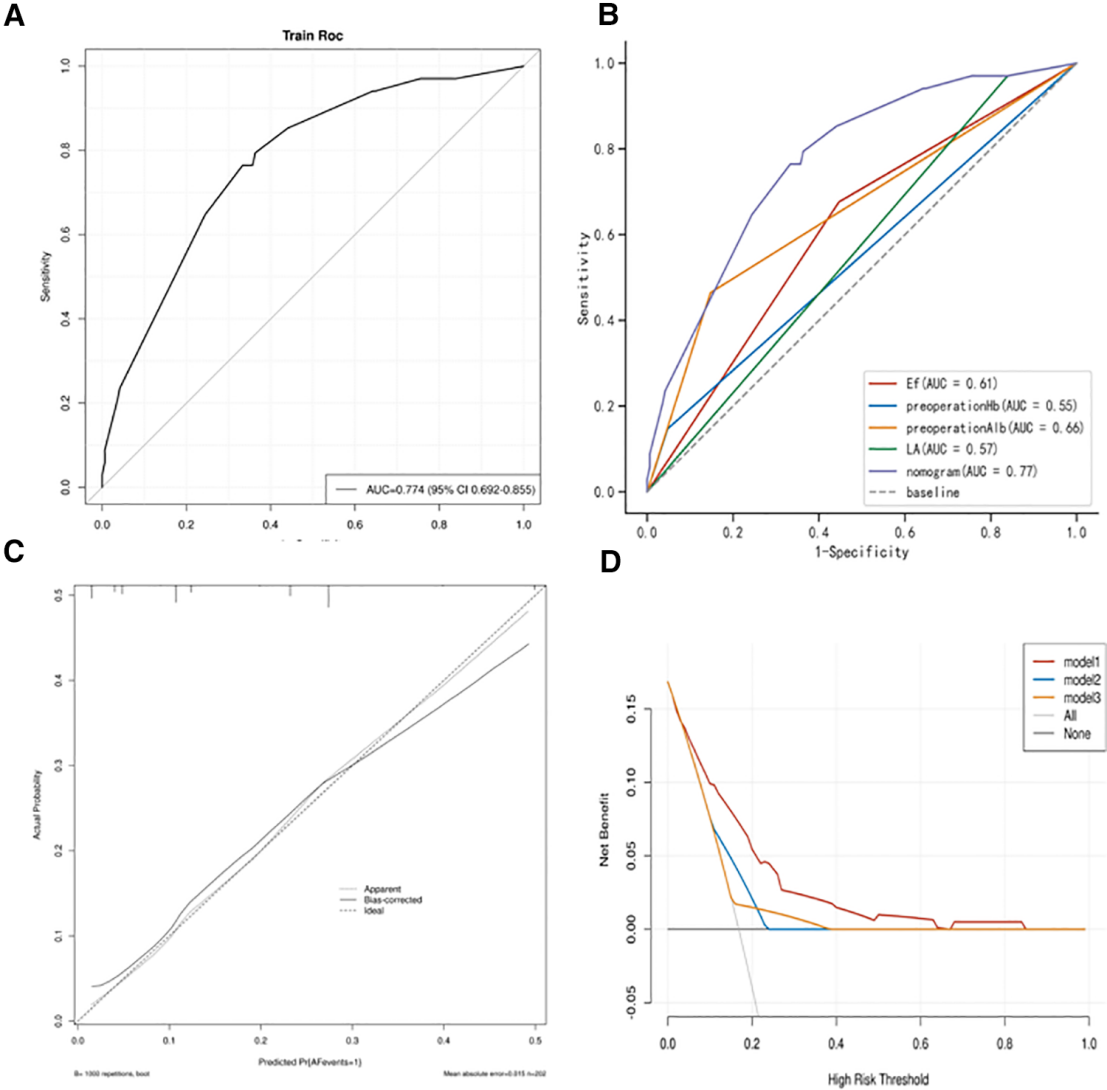
(**A**) Receiver operating characteristic (ROC) curves of the established nomogram model; (**B**) Comparison of ROC curves between the established nomogram model and the constructed factors; (**C**) Calibration curve of the established nomogram model; (**D**) Decision curve analysis of the established nomogram model.

We used the independent risk factors determined by the LASSO logistic regression strategy; we developed a nomogram model to predict POAF (Figure 3). The AUC of the established nomogram model was 0.76 (95% CI 0.69–0.86), which indicated the good discriminative ability of the model (Figure 4A). In addition, the AUC of the nomogram model was superior to each factor included in the model (Figure 4B). The Briser score of the calibration curve was 0.12, which indicated that the predicted results were highly consistent with the actual results (Figure 4C). The DCA indicated that this nomogram model had a high clinical application value (Figure 4D).

## Discussion

POAF is a common complication after esophagectomy, and the overall incidence of POAF in this study was 16.83% (34/202). Compared with non-esophageal surgery, patients undergoing esophagectomy had a higher incidence of POAF (17.66% vs 7.63%) (13). There was a significant increase in postoperative hospital stay (*p* = 0.04), hospital expenses (*p* = 0.01), and CCI (*p* < 0.001). Therefore, the identification of independent risk factors and the development of an accurate predictive model for POAF are critical for optimal treatment planning in high-risk individuals with MIE after neoadjuvant therapy. Preoperative Alb ≦41.2 g/L, LA diameter >32.9 mm, preoperative Hb >149 g/L, and EF >67.61% were identified as the independent risk factors for POAF, and a novel nomogram model was constructed to predict POAF. The model not only showed the good discriminative ability but also had the best agreement between the predicted results and the observed results. Based on this nomogram model, each prognostic factor was quantified and visualized with a nomogram model to predict the probability of POAF. By using this predictive nomogram, physicians could judge individual risk, predict outcomes, personalize treatment, and take preventive measures for patients at high risk.

In this study, we determined LA diameter ≥32.9 mm as an independent risk factor of POAF. Nagatsuka et al. investigated 200 patients undergoing esophagectomy for EC and determined a LA diameter ≥36.0 mm [odds ratio (OR) 2.47, 95% CI 1.06–5.71] as an independent risk factor (*p* = 0.035) (14). A relationship between LA diameter and AF has been proposed in the general population. One hypothesized direct underlying cause of AF is the result of organic changes in the “remodeling” of the left atrium to maintain a normal sinus rhythm. Increased left ventricular diastolic blood pressure during diastolic dysfunction is associated with increased left ventricular diastolic blood pressure. With the increase of left atrial pressure, atrial wall extension increases and atrial remodeling occurs (15). Interestingly, we also found that left ventricular EF >67.61% was an independent risk factor of POAF. This finding seemed to be inconsistent with previous reports. Zacharias et al. enrolled a total of 8,051 consecutive cardiac surgery patients and found that EF <40% (OR 1.16, 95% CI 1.03–1.31) was an independent risk factor of POAF (16). However, a large cohort study (203,135 patients from Pennsylvania and 35,976 patients from New Zealand) investigated the relationship between left ventricular ejection fraction (LVEF) and mortality, and they found an HR of 1.71 (95% CI 1.64–1.77) at LVEF of ≥70% and an HR of 1.73 (95% CI 1.66–1.80) at LVEF of 35%–40%, which indicated a U curve relation between LVEF and mortality (17). Another analysis of 2,867 ICU patients (including 324 patients with EF >70%) showed that the presence of EF >70% increased 28-day mortality (OR 1.39, 95% CI 1.04–1.84) (18). This finding first suggested the association between the high LVEF and the POAF. Further studies are necessary to explore the mechanisms.

There are still limited studies focusing on the relationship between preoperative serum Alb and POAF among patients undergoing esophagectomy. Zhong et al. explored the association between serum Alb and paroxysmal AF based on a Chinese cohort of 305 patients with AF and 610 patients without AF and found that low Alb in male patients is a risk factor for paroxysmal AF (19). Liao et al. conducted a large-scale epidemiological and Mendelian randomization (MR) study and found that the serum Alb level was negatively correlated with the incidence of AF, but the causal relationship between serum Alb level and AF was not clarified (20). In this study, we found that preoperative Alb ≦41.2 g/L was associated with a higher incidence of POAF. This finding supports that low Alb contributed to the occurrence of POAF. Serum Alb plays important roles in anti-inflammatory, antioxidant, anticoagulant, antiplatelet aggregation, and colloid osmotic effects. One recent dose–response analysis showed that for each increase of 10 g/L in serum Alb, the risk of AF would decrease by 36% (21). Present evidence supports that hypoalbuminemia is a modifiable risk factor associated with cardiovascular events (22). In future studies, it would be interesting to explore the relationship between preoperative nutrition and the incidence of POAF among patients with ESCC. Similarly, there are still no reports investigating the relationship between high Hb and POAF among patients with ESCC. Recently, Nakatani et al. found that high Hb is an independent risk factor of new-onset AF among patients with heart failure with preserved EF (23). Commonly, patients with paroxysmal AF often have elevated Hb in clinical practice (24). One explanation was that polyuria induced by the excess secretion of atrial natriuretic peptide contributed to the high Hb in patients with AF.

At present, there are different opinions on whether to take preventive treatment for POAF (25). Rao et al. held the opinion that the simple prevention of POAF, including using prophylactic drugs, was unlikely to improve long-term survival and unlikely to be cost-effective (11). However, the model including age and neoadjuvant therapy established by Rao et al. only had a moderate c-statistic (0.62). Compared with previous models, the nomogram model in this study had an AUC of 0.76, which indicated a better discriminative ability. Therefore, we suggest taking measures to prevent the occurrence of POAF when the nomogram model suggests a high possibility of POAF. Although AF can occur as an isolated event, it can occur in conjunction with other complications in a population predisposed to cardiopulmonary complications. The application of enhanced recovery after surgery is necessary to reduce overall mortality and morbidity.

To the best of our knowledge, this study was the first predictive nomogram model for POAF in patients with ESCC receiving neoadjuvant therapy. However, the study has the following limitations: first, the model was analyzed based on retrospective data, which may have a potential bias due to a lack of randomization, patient selection, and some missing values. Second, although nCRT is currently the first choice for patients with low events raised by the radiotherapy, relatively few patients received nCRT in this cohort. Further, we did not conduct a subgroup analysis to evaluate the effect of radiation dose on the incidence of POAF. Third, the prediction model has good discrimination, but it has not been verified externally. Further, the case number is relatively limited. External validation is necessary before applying the nomogram model to patients at other centers. Four, whether this nomogram is suitable in patients with locally advanced esophageal adenocarcinoma remains unclear.

## Conclusions

In summary, we determined preoperative Alb ≦41.2 g/L, LA diameter >32.9 mm, preoperative Hb >149 g/L, and EF >67.61% to be the risk factors for POAF among patients with ESCC receiving neoadjuvant therapy and MIE. A novel and useful nomogram model was constructed and validated to help clinicians evaluate the risk of POAF and take personalized treatment plans. The predictive ability and clinical value of the nomogram model were promising. For additional external validation, generalization, and application of this prediction model, large prospective multicenter studies are needed.

## Data Availability

The original contributions presented in the study are included in the article/Supplementary Material, further inquiries can be directed to the corresponding author.
